# Peripapillary Retinal Nerve Fiber Layer Changes in Preclinical Diabetic Retinopathy: A Meta-Analysis

**DOI:** 10.1371/journal.pone.0125919

**Published:** 2015-05-12

**Authors:** Xiaofei Chen, Chuang Nie, Yan Gong, Ying Zhang, Xin Jin, Shihui Wei, Maonian Zhang

**Affiliations:** Department of Ophthalmology, Chinese PLA General Hospital, Beijing, China; Queen's University Belfast, UNITED KINGDOM

## Abstract

**Background:**

Diabetic retinopathy is a microvascular neurodegenerative disorder in diabetic patients. Peripapillary retinal nerve fiber layer changes have been described in patients with preclinical diabetic retinopathy, but study results have been inconsistent.

**Objective:**

To assess changes in peripapillary retinal nerve fiber layer thickness in diabetic patients with preclinical diabetic retinopathy.

**Methods:**

A literature search was conducted through PubMed, EMBASE, Web of Science and Cochrane Library. Case-control studies on RNFL thickness in preclinical diabetic retinopathy patients and healthy controls were retrieved. A meta-analysis of weighted mean difference and a sensitivity analysis were performed using RevMan 5.2 software.

**Results:**

Thirteen case-control studies containing 668 diabetic patients and 556 healthy controls were selected. Peripapillary RNFL thickness was significantly reduced in patients with preclinical diabetic retinopathy compared to healthy controls in studies applying Optical Coherence Tomography (-2.88μm, 95%CI: -4.44 to -1.32, P = 0.0003) and in studies applying Scanning Laser Polarimeter (-4.21μm, 95%CI: -6.45 to -1.97, P = 0.0002). Reduction of RNFL thickness was significant in the superior quadrant (-3.79μm, 95%CI: -7.08 to -0.50, P = 0.02), the inferior quadrant (-2.99μm, 95%CI: -5.44 to -0.54, P = 0.02) and the nasal quadrant (-2.88μm, 95%CI: -4.93 to -0.82, P = 0.006), but was not significant in the temporal quadrant (-1.22μm, 95%CI: -3.21 to 0.76, P = 0.23), in diabetic patients.

**Conclusion:**

Peripapillary RNFL thickness was significantly decreased in preclinical diabetic retinopathy patients compared to healthy control. Neurodegenerative changes due to preclinical diabetic retinopathy need more attention.

## Introduction

Diabetic retinopathy is a retinal vascular lesion in patients with diabetic mellitus[1]. Studies have indicated that neurodegenerative changes have also been found in the retina of diabetic patients, including apoptosis of retinal neuronal cells and activation of glial cells [2–4]. In addition, previous clinical studies have found impairment of visual functions, such as contrast sensitivity and color vision, as well as electrophysiological changes in diabetic patients with early diabetic retinopathy[5–9]. Recently, defects in Humphrey Matrix testing and multifocal electroretinograms, both of which are associated with retinal neuronal dysfunction, have been described in diabetic patients without visible vascular changes in the retina[10,11].

Axons of retinal ganglion cells compose the retinal nerve fiber layer (RNFL) in the retina and then form the optic nerve connecting the eyeball and brain. Retinal nerve fiber layer (RNFL) loss is recognized as an important neurodegenerative sign in glaucoma[12]. Thinning of the RNFL has also been found in multiple sclerosis[13], Parkinson’s disease[14] and Alzheimer’s disease[15], indicating neurodegeneration of the retina. In recent years, several studies have indicated occurrence of peripapillary RNFL thinning in the retina of diabetic patients without detectable diabetic retinopathy[16,17], while the difference of RNFL thickness between diabetic patients and healthy controls was not significant in other studies[18,19]. If RNFL thinning is significant in diabetic patients with preclinical diabetic retinopathy, evaluation of peripapillary RNFL thickness would be very important, because early detection and treatment of diabetic retinopathy is critical to reduce the risk of blindness[20]. To address this issue, a systemic review and meta-analysis of studies investigating peripapillary RNFL thicknesses of diabetic patients without clinical diabetic retinopathy and healthy controls were performed.

## Materials and Methods

### Search strategy

Databases including PubMed, EMBASE, Web of Science and the Cochrane Library were searched using the terms “diabetes mellitus”, “retinal nerve fiber layer” and “RNFL” up to February 24^th^, 2015. Language and location were not restricted. References lists of all included studies were also carefully checked.

### Study Selection

Studies that fulfill the following criteria were included for meta-analysis: 1. healthy controls were included; 2. patients had diabetes mellitus; and 3. thickness of the peripapillary RNFL was measured. Studies were excluded for anyone of the following reasons: 1. peripapillary RNFL thickness was not quantitatively measured; 2. a subgroup of patients without clinical diabetic retinopathy (NDR) was not included; 3. both eyes were used for statistical analysis; and 4. data of RNFL thickness were not eligible for analysis. Two reviewers (C.X.F and Z.M.N) evaluated each study based on the above criteria and disagreements were solved by discussion.

### Data Extraction

Data were retrieved by two reviewers (C.X.F and N.C) independently including first author, year of publication, location, number of subjects, type of diabetes, duration of diabetes, mean age, gender, level of HbA1c, type of measuring instrument, and average peripapillary RNFL thickness in total and in four quadrants (superior, inferior, temporal and nasal). Discrepancies were discussed until an agreement was reached.

### Quality Assessment

The Newcastle-Ottawa Scale (NOS) was used to evaluate the method quality of included studies[21]. The selection criteria of subjects, comparability between controls and cases and outcomes of each study were assessed with scores raging from 1 to 9. A score of 6 or higher was considered to be of relatively higher quality. The assessment was conducted by two reviewers (C.X.F and N.C) and differences were discussed until an agreement was reached.

### Statistical Analysis

Cochrane Collaboration’s Review Manager Software (RevMan 5) was used for data analysis. Means and standard deviations of the RNFL thickness values were obtained as continuous variables to calculate the weighted mean difference. Heterogeneity of the included studies was tested by Chi-square test and Higgins I^2^ test. The fixed-effect analysis model was applied if the heterogeneity was not significant (P>0.10, I^2^<50%). Otherwise, the random-effect analysis model was used (P≤0.10, I^2^≥50%). Publication bias was evaluated by Funnel plot. Sensitivity analysis was conducted by sequentially excluding one study each time and recalculating weighted mean difference of RNFL thickness of the remained studies. A p value<0.05 was considered significant.

## Results

### Characteristics and Quality of Selected Studies

**Fig 1:**
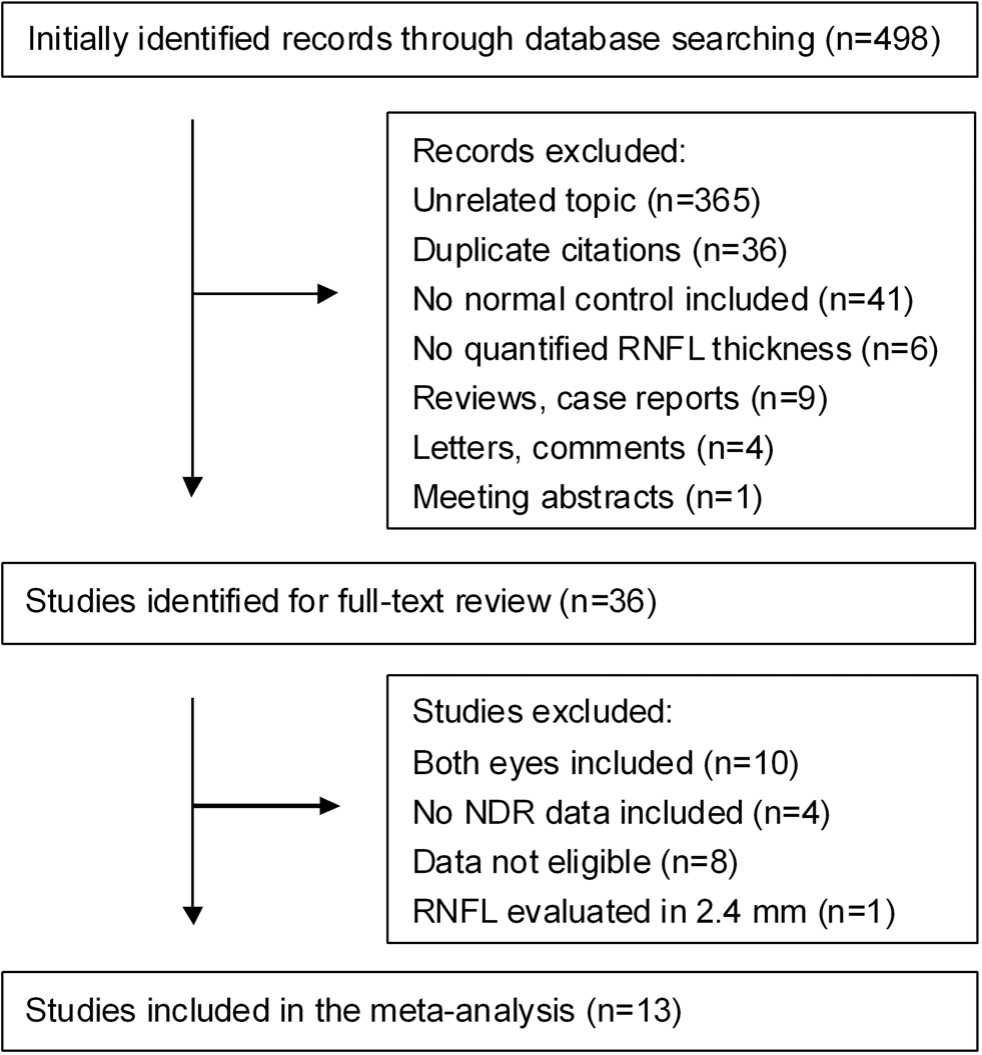
Process of study selection. NDR, diabetic patients without clinical diabetic retinopathy.

In the records initially identified, RNFL thickness was quantitatively evaluated by Optical Coherence Tomography (OCT), Scanning Laser Polarimeter (SLP) containing GDx with fixed corneal compensator mode (GDx NFA) and GDx with variable corneal compensator mode (GDx VCC) or Heidelberg Retina Tomography (HRT). The selection process is shown in Fig 1. During selection, one study using OCT was excluded because a smaller scanning circle of 2.4 mm in diameter was applied in this study, while a scanning circle of 3.4–3.46 mm in diameter was applied in other studies[22]. Two studies using HRT were excluded because no subgroup of diabetic patients without clinical diabetic retinopathy was analyzed[23,24]. Finally, 13 studies [10,16–19,25–32] applying OCT or SLP for RNFL evaluation were included in our study.

The included studies were published from 2002 to 2014, with 1 each from Greece, Italy, Brazil, Korea, Taiwan, Hong Kong, and 2 each from Japan and Mainland China and 3 from Turkey. The characteristics and quality of studies are shown in Table 1.

**Table 1:** Characteristics and quality of selected studies.

Study	Location	Type of DM	No. of eyes		Age (years)		Gender (M/F)		Duration of DM (years)	HbA1c (%)	Instrument	Study quality
			DM	HC	DM	HC	DM	HC				
**Xin (2014)**	China	2	48	30	57	56	25/23	16/14	6.1±2.3	6.7±1.0	3D-OCT	6
**Ma (2013)**	China	2	96	115	51.1	58.6	51/45	60/55	33.75±9.11*	NR	SD-OCT	6
**Lung (2012)**	Hong Kong	2	10	14	51	49.4	NR		6.9±6.9	NR	Stratus OCT	5
**Gonul (2011)**	Turkey	1	98	49	17.02	18.71	54/44	20/29	60.76±50.41*	NR	Stratus OCT	5
**Park (2011)**	Korea	NR	37	40	66.2	64.3	15/22	21/19	12.3±3.3	4.4±2.3	Cirrus OCT	6
**Oshitari (2009)**	Japan	2	45	30	61.6	60.0	25	16	4.8±4.4	7.4±1.9	Stratus OCT	5
**Peng (2009)**	Taiwan	1&2	99	77	56.9	55.2	50/49	30/47	6.2±4.4	8.3±1.8	Stratus OCT	6
**Sugimoto (2005)**	Japan	2	32	34	55.4	50.9	NR		7.9±7.3	6.90±1.75	OCT	6
**Takis (2014)**	Greece	2	27	25	65.4	62.8	10/17	10/15	12.8±5.9	7.2±1.6	SLP	6
**Parravano (2008)**	Italy	1	30	30	36.77	35.87	11/19	9/21	12.23±10.83	7.38±1.19	SLP	6
**Lonneville (2003)**	Turkey	2	40	50	54.9	51.2	14/26	22/28	98.7±92.1*	11.8±1.0	SLP	5
**Lopes (2002)**	Brazil	1	12	12	30	29	4/8	4/8	14±5	6.9±2.3	SLP	7
**Ozdek (2002)^#^**	Turkey	2	50	50	50.8	51.2	NR		37.7±43.5*	6.6±0.8	SLP	5
			44		49.8				64.9±64.3*	9.5±1.4		

DM, diabetic mellitus; HC, healthy controls; NR, not reported;

* Duration of DM (months);

# Two groups of diabetic patients without diabetic retinopathy included in the study.

### Meta-analysis

**Fig 2:**
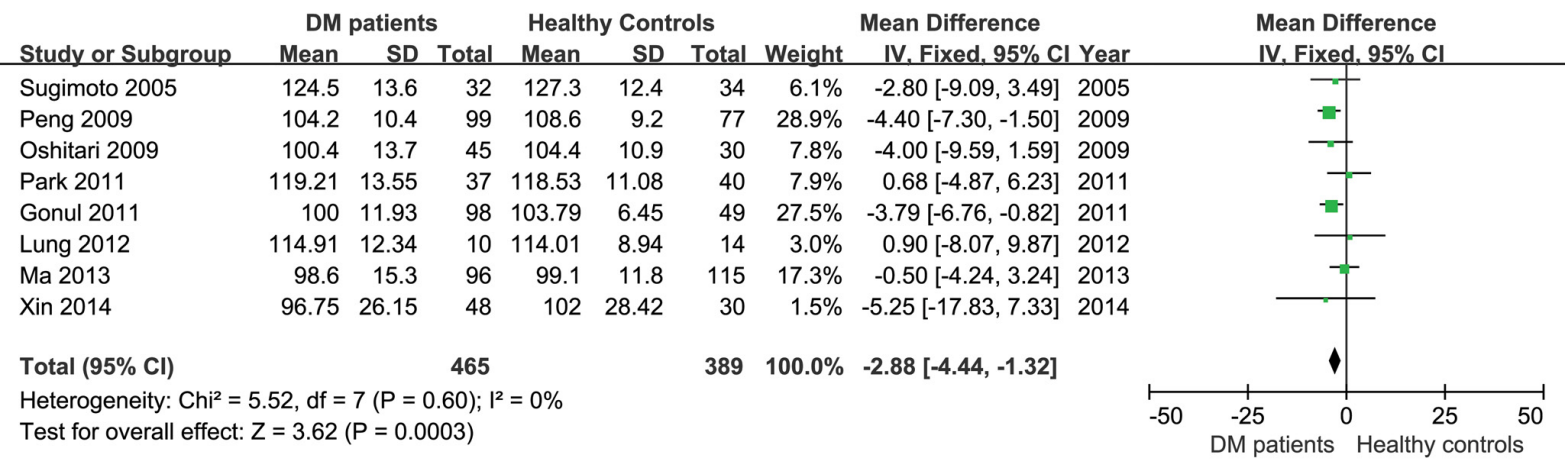
Meta-analysis of average RNFL thickness of diabetic patients and healthy controls in studies using OCT.

**Fig 3:**
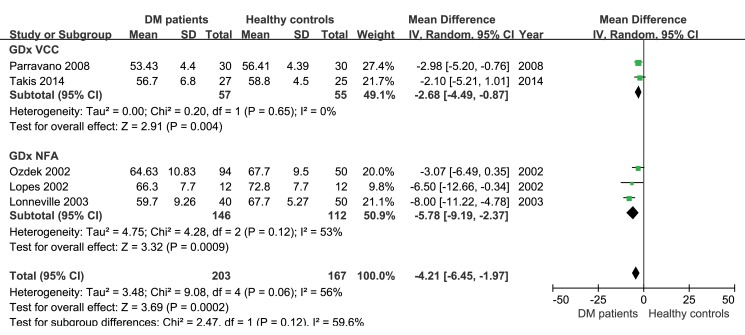
Meta-analysis of average RNFL thickness of diabetic patients and healthy controls in studies using SLP.

**Fig 4:**
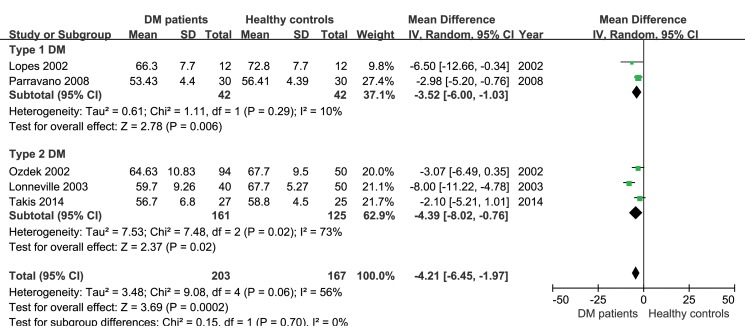
Subgroup analysis of average RNFL thickness of type 1 and type 2 diabetic patients.

The peripapillary RNFL thickness difference between diabetic patients without diabetic retinopathy and age-matched healthy controls were analyzed separately in studies using OCT and studies using SLP, because the values obtained through the two instruments were not comparable. In the 8 studies applying OCT, the heterogeneity of the included studies was not significant (P = 0.60, I^2^ = 0%), therefore, the fixed-effect analysis model was used. The results of a meta-analysis showed that peripapillary RNFL thickness in diabetic patients without diabetic retinopathy was significantly less than that in age-matched healthy controls (-2.88μm, 95%CI: -4.44 to -1.32, P = 0.0003, Fig 2). Meanwhile, analysis of the 5 studies applying SLP also showed reduced peripapillary RNFL thickness (-4.21μm, 95%CI: -6.45 to -1.97, P = 0.0002, Fig 3) in diabetic patients without diabetic retinopathy. A subgroup analysis based on the type of diabetes was also performed in studies using SLP. The results indicated that average RNFL thickness decreased both in type 1(-3.52μm, 95%CI: -6.00 to -1.03, P = 0.006, Fig 4) and type 2 (-4.39μm, 95%CI: -8.02 to -0.76, P = 0.02, Fig 4) diabetic patients.

**Fig 5:**
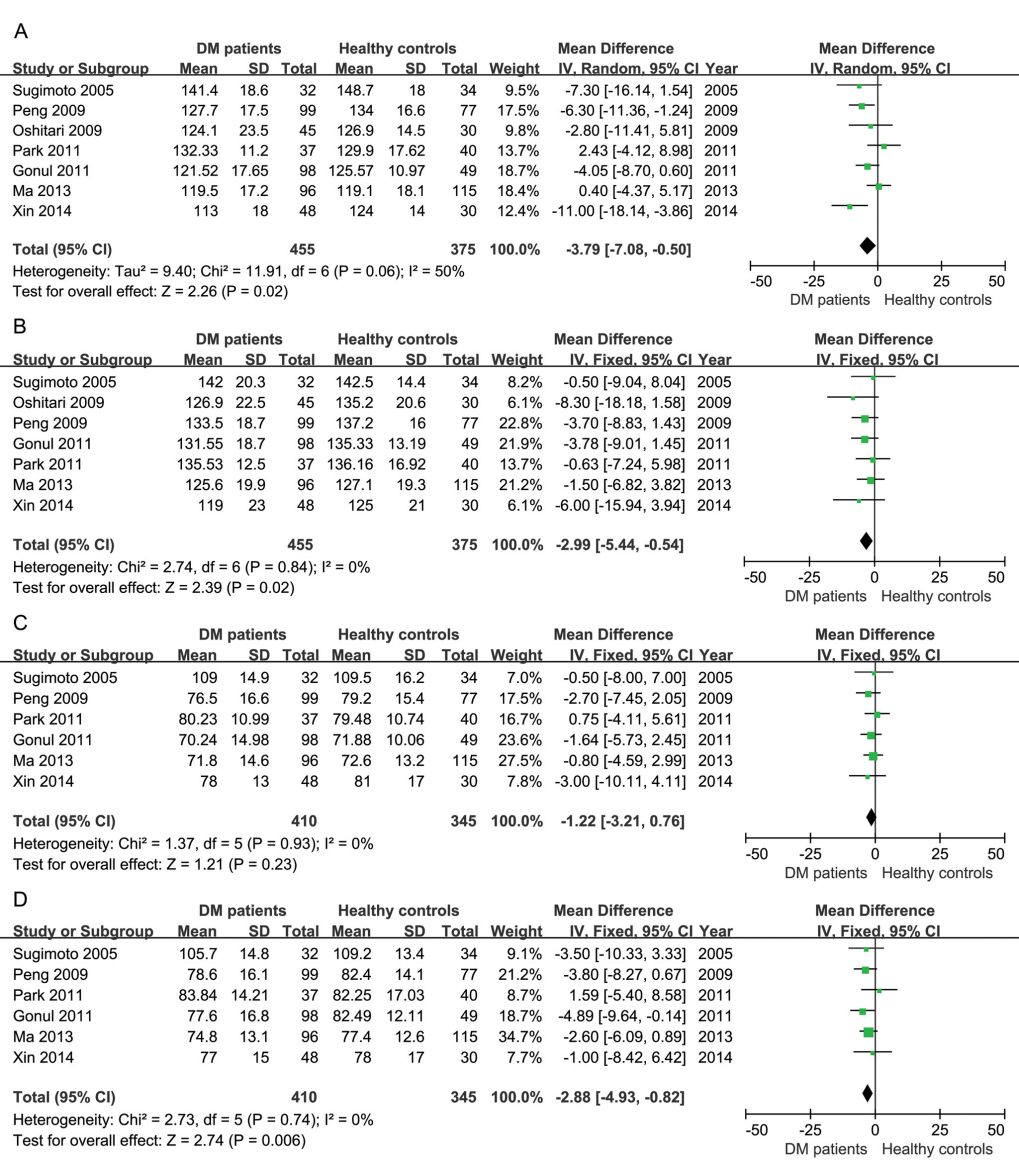
Meta-analysis of RNFL thickness in four quadrants in studies using OCT. A, superior; B, inferior; C, temporal; D, nasal.

The peripapillary RNFL thickness in four quadrants was also analyzed in the studies using OCT. As shown in Fig 5, RNFL thicknesses in the superior quadrant (-3.79μm, 95%CI: -7.08 to -0.50, P = 0.02), inferior quadrant (-2.99μm, 95%CI: -5.44 to -0.54, P = 0.02) and nasal quadrant (-2.88μm, 95%CI: -4.93 to -0.82, P = 0.006) of the retinas in diabetic patients were significantly reduced, but thickness reduction was not observed in the temporal quadrant (-1.22μm, 95%CI: -3.21 to 0.76, P = 0.23).

**Fig 6:**
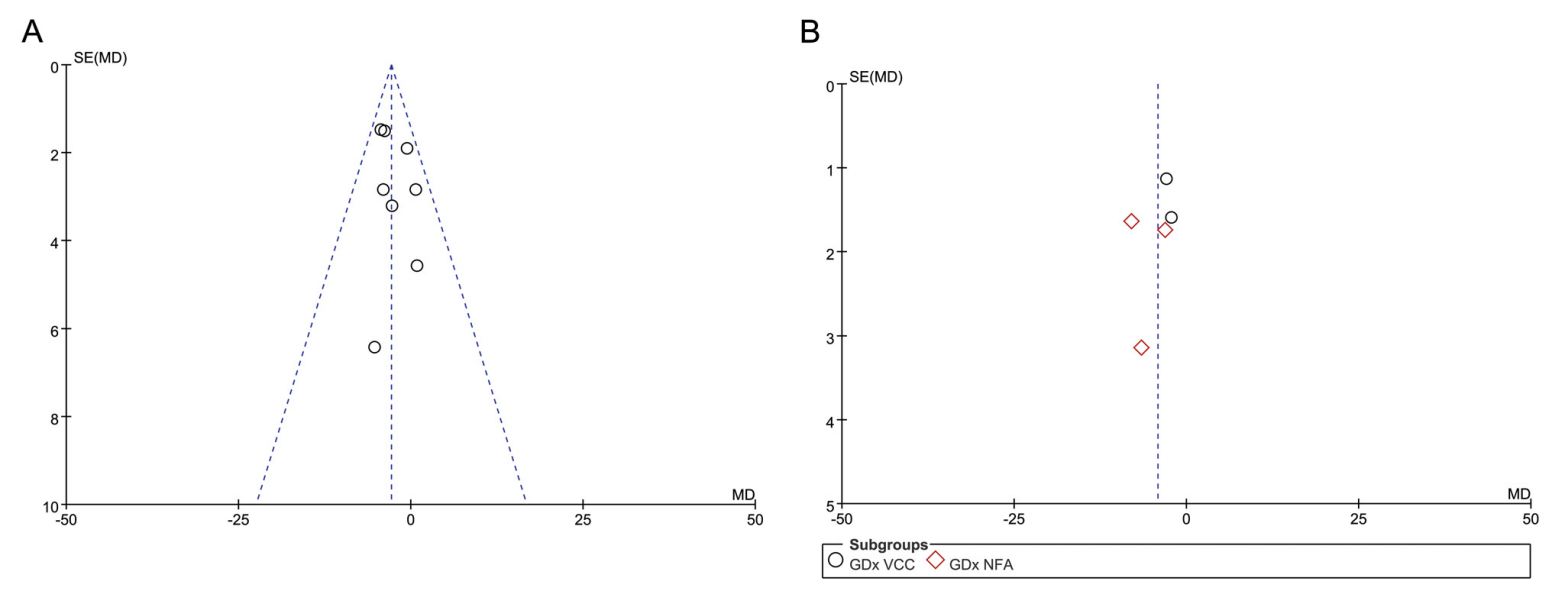
Funnel plot of all selected studies using OCT and studies using SLP. A, Funnel plot of studies using OCT; B, Funnel plot of studies using SLP.

**Fig 7:**
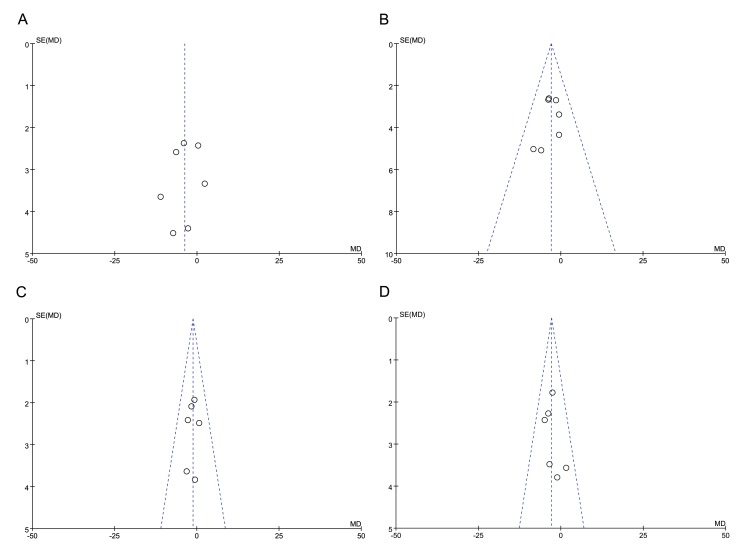
Funnel plot of studies assessing RNFL thickness in four quadrants using OCT. A, superior; B, inferior; C, temporal; D, nasal.

No obvious publication bias was identified by funnel plots in studies to assess average RNFL thickness in total and in four quadrants (Fig 6 and Fig 7). A sensitivity analysis was performed in studies using OCT and in studies using SLP. The results indicated that the reduction of RNFL thickness in diabetic patients was still significant no matter which one was excluded in studies using OCT or SLP (Table 2).

**Table 2:** Results of sensitivity analysis.

	Fixed-effect analysis		Random-effect analysis		Heterogeneity
Study excluded	Mean difference (μm, 95%CI)	p value	Mean difference (μm, 95%CI)	p value	*I* ^*2*^ (%)
**Xin (2014)**	-2.84 (-4.41 to -1.27)	0.0004	-2.84 (-4.41 to -1.27)	0.0004	0
**Ma (2013)**	-3.38 (-5.09 to -1.67)	0.0001	-3.38 (-5.09 to -1.67)	0.0001	0
**Lung (2012)**	-3.00 (-4.58 to -1.42)	0.0002	-3.00 (-4.58 to -1.42)	0.0002	0
**Gonul (2011)**	-2.54 (-4.37 to -0.71)	0.007	-2.54 (-4.37 to -0.71)	0.007	0
**Park (2011)**	-3.18 (-4.81 to -1.56)	0.0001	-3.18 (-4.81 to -1.56)	0.0001	0
**Oshitari (2009)**	-2.79 (-4.41 to -1.16)	0.0008	-2.79 (-4.41 to -1.16)	0.0008	0
**Peng (2009)**	-2.26 (-4.11 to -0.42)	0.02	-2.26 (-4.11 to -0.42)	0.02	0
**Sugimoto (2005)**	-2.89 (-4.49 to -1.28)	0.0004	-2.89 (-4.49 to -1.28)	0.0004	0
**Takis (2014)**	-4.41 (-5.97 to -2.84)	0.00001	-4.83 (-7.52 to -2.14)	0.0004	59
**Parravano (2008)**	-4.57 (-6.36 to -2.77)	0.00001	-4.71 (-7.75 to -1.68)	0.002	62
**Lonneville (2003)**	-3.00 (-4.55 to -1.45)	0.0001	-3.00 (-4.55 to -1.45)	0.0001	0
**Lopes (2002)**	-3.80 (-5.24 to -2.37)	0.00001	-3.97 (-6.44 to -1.50)	0.002	64
**Ozdek (2002)**	-4.12 (-5.64 to -2.59)	0.00001	-4.57 (-7.42 to -1.71)	0.002	66

## Discussion

The data of this study showed that the average peripapillary RNFL thickness in diabetic patients without clinical diabetic retinopathy was significantly decreased compared to age-matched healthy controls.

In the selected studies, a circular circumpapillary scan of 3.4–3.46 mm in diameter was applied by OCT to calculate RNFL thickness, while a calculation circle with 2.4 mm in inner diameter and 3.2 mm in outer diameter was used by GDx VCC and an ellipse at 1.75 disc diameter was used by GDx NFA. Although different peripapillary target areas were measured by OCT and SLP, analysis showed that the results were consistent. In addition, two studies using HRT, that evaluated RNFL thickness at the edge of optic disc, also showed reduced peripapillary RNFL thickness in diabetic patients[23,24].

In the study of Takahashi et al[33], both stratus OCT and GDx VCC were applied to detect RNFL loss in patients with mild to moderate diabetic retinopathy and glaucoma, and RNFL thinning in diabetic patients was detected by GDx VCC but not by OCT. The authors suggested the difference was caused by fluid accumulation in the retina due to relatively severe diabetic retinopathy in this study and involvement of Müller cells and astrocytes during RNFL measurement by OCT[34]. The researchers also speculated that the difference could be not obvious when evaluating RNFL in diabetic patients without diabetic retinopathy. However, in other studies, RNFL thinning was accelerated by the progression of diabetic retinopathy[16,35].

Retinal nerve fiber loss was considered to be associated with retinal ganglion cell dysfunction and apoptosis. In diabetic animal models, damage of retinal neuronal cells and inner retinal thinning have been detected in the early stage of diabetic retinopathy[36,37]. Neuronal apoptosis in the retina of diabetic patients has also been reported by Barber et al[3]. Both anterograde and retrograde axonal transportation was compromised in diabetic animals, most likely because of damaged polyol metabolism and impaired mitochondria function of retinal ganglion cells[38,39]. The cross-sectional size of large optic nerve fibers has also been found to decrease in diabetic rats[40].

In addition, obvious accumulation of advanced glycation end products (AGEs) in the cribriform plates, connective tissues and around vessels of the optic nerve of diabetic patients has been reported[41]. AGEs have been considered contributing to dysfunction of intracellular anti-oxidant enzymes, transcription factors and mitochondrial proteins, as well as impairment of elastic property of the extracellular matrix and cribriform plates[42,43].

Other than the initial neuronal degenerative changes, microangiopathy caused by diabetes in the optic nerve head has also been attributed to the RNFL defection. Chihara et al.[44] reported RNFL loss in diabetic patients through fundus photograph and speculated that cotton-wool spots, which are caused by microvascular nonperfusion and sometimes fade without visible signs in the retina, may attribute to the damage of retinal nerve fibers. Nevertheless, the pathophysiological microvascular changes of diabetic retinopathy, including endothelium dysfunction, vascular basement membrane thickening, pericyte apoptosis and capillary occlusion, may also involve capillary vessels in the optic nerve[45–48].

A coorelation between blood glucose (BG) control and RNFL defects has not been shown. In a previous study, the mean superior maximum of RNFL was reduced in BG-non-regulated diabetic patients without diabetic retinopathy, but not in BG-regulated diabetic patients[32]. In another study, none of the GDx variables of RNFL were changed in diabetic patients after one-month of BG regulation[30]. Additional information on metabolic parameters is needed to further investigate reasons for different results among these studies. A multiple-center and large-scale study with higher method quality is necessary.

Some limitations exist in this meta-analysis. First, although there was no restriction in language, location, or evaluation methods during the search in databases, the included studies that applied OCT for RNFL measurement were all conducted in Asia, while the included studies applying SLP were performed in Europe, South America and Asia. No studies using HRT were included. Second, in several studies, method quality, as assessed by NOS, was relatively low, mainly because the representativeness of cases and controls, as well as blinding methods, was not clarified. Third, the type of diabetes mellitus was not considered in some studies. Additionally, the age, duration of disease, and level of HbA1c were different among included studies, all of which could be confounding factors during meta-analysis.

In conclusion, peripapillary RNFL thickness was significantly reduced in diabetic patients without detectable diabetic retinopathy. Measurement of peripapillary RNFL thickness may become a novel way to evaluate and monitor early retinal changes in diabetic patients. Multicenter studies with larger population are still necessary to assess the efficacy and importance of this measurement. Nevertheless, neurodegenerative changes of retina and corresponding visual functional impairment in diabetic patients need more attention during clinical evaluation of diabetic retinopathy.

## Supporting Information

S1 Checklist PRISMA Checklist. (DOC)

S1 Diagram PRISMA Flow Diagram. (DOC)

S1 Text Search Strategy and Records from EMBASE. (TXT)
