# Supplementary material for: Peripapillary Retinal Nerve Fiber Layer Changes in Preclinical Diabetic Retinopathy: A Meta-Analysis
Source: PLoS One. 2015 May 12;10(5):e0125919. doi: 10.1371/journal.pone.0125919 (PMC4429076; doi:10.1371/journal.pone.0125919)
Supplement: S1 Diagram — (DOC) [file pone.0125919.s002.doc]

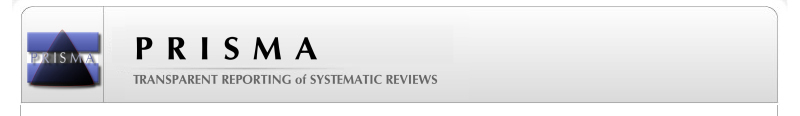
**PRISMA 2009 Flow Diagram**

**Screening**

**Included**

**Eligibility**

**Identification**

Records identified through database searching
(n = 498 )

Records after unrelated topic excluded
(n = 133 )

Records screened
(n = 97 )

Duplicates removed
(n = 36 )

Full-text articles assessed for eligibility
(n = 36 )

Full-text articles excluded:

No normal control included (n=41)

No quantified RNFL thickness (n=6)

Reviews, case reports (n=9)

Letters, comments (n=4)

Meeting abstracts (n=1)

Studies included in qualitative synthesis
(n = 21 )

Studies included in quantitative synthesis (meta-analysis)
(n = 13 )
